# Remarks on Reviewing

**Published:** 1809-12

**Authors:** 


### *To the Editors of the Medical and Physical Journal.*

Gentlemen,

Reviews are become a necessary evil. It is very useful to resort to these literary jackalls for a faithful report of the prey they hunt down, and judge at our leisure of the flavour of the food we would be inclined to select, without the risk of appropriating to ourselves such rank and good-for-nothing game, as would be little accommodated to a delicate palate.

But if we cannot confide in the nose of our jackalls, or if they set up a yell out of time or tune to mislead us, it would be much better to discard them in toto, and trust to our own guidance, though at the expence of a little more labour, money, and time.

To come nearer the point, I have no quarrel with the

Medical

Medical Review of another learned metropolis; nor indeed with your own ingenious critiques, candid and gentle Editors of the London Medical Journal, as I more forcibly evince by my selecting you as my organ on this occasion, than I could by the most overpowering effulgence of compliment. But there is one of the trade that has been quarterly leading me astray, and perhaps, many other ignoramusses like myself, for the last twenty months; but I have detected the gentleman's unfitnes for the office he administers, and I think it but honest to make known my discovery, in hopes of keeping out of ditches, other honest simple folks, who might be inclined to trust themselves to *such guides*.

His last number convinces me, that he entertains an utter aversion to every improvement that could shorten or better his road, or turn out of advantage to his fellow travellers in this sublunary pilgrimage. In a word, as some people shrink from a cat, or perspire at cheese, this impartial Reviewer shudders at talent and genius, and this well founded antipathy is the standard by which he measures the length, breadth, and depth of every delinquency or perfection that comes under his inspection.

The first ebullition of this aversion effervesces in his eulogium of Mr. Pearson's "Principles of Surgery," from which he quotes a passage, "possessing more than common strength and brilliancy, and speaking *the critic's* sentiments far more forcibly than he could speak them for himself." The passage which he esteems so congenial with his own sentiments, is a judicious remark, that, "Inattention to the scientific part of surgery, not uncommonly arises from the *conceit* of superior talents; and that nothing is more flattering to youthful vanity, nor inimical to his progress in knowledge, than for a young man to *fancy* himself to be a genius." Little, however, did this writer perceive that Mr. Pearson was not possessed, like himself, of a raging mania against talent, and that the name of John Hunter, and some others, did not throw him into convulsions, but that his only objection lay against the *conceit* and *fancy* of superior genius, and not against genius itself.

In other matters, there is not the slightest difference of opinion between the Critic and Mr. Pearson. Every thing and every word is "masterly," and "eloquent," and "modest," and "wise," and "judicious;" and in the desiderata of "Observation," "Philosophy," "Investigation and Induction," he *shrewdly* questions whether Mr. Pearson has

 a rival a rival in the profession at large. Lastly, a chapter in which "some *objections* are made to the old divisions of cancer," and in which cancers of different parts are *described* with an accuracy that denotes much and minute observation, and in which, "the desiderata in the *treatment* naturally suggest themselves to every benevolent mind," is dignified by the title of *a valuable chapter.*

Mr. Pearson is a man of high eminence in his profession, and of great and long established character, otherwise all this sickening panegyrick which his good sense must despise, would never have been lavished on him.

For the same reason, Mr. Davy also comes in for his share of applause.—It is now too late to be inimical to his talents, and it may induce a character of candour to appear to do them justice. But this writer, not contented with bestowing on his elegant and invaluable discoveries, their appropriate praises, confuses himself and his reader in a huddle of fine words, about "a competition with Sir Isaac Newton's philosophic greatness," and "a dazzling and elevated position which admits of *one advancement only in the ascent of honour,*" the meaning of which I should have great satisfaction in discovering if I could.

Poor Dr. Lambe may be ripe, for aught I know, for a "mad house," and Dr. Arnold for a "conventicle."—Dr. Reeve may be very properly condoled with on "his discreditable publication," and Monsieur Richerand abused for his silly opinion, that French surgeons are as good as British; yet if those gentry had been rich, substantial, elderly, and even stupid well-established *English* practitioners, they would probably have been sucking, at this moment, the sweet honey-comb of applauses, profusely crammed down their throats by the compilation of the work alluded to.

I have strong reason to believe, that there is not one of the group, of whom I would not form a different opinion from that recommended to my adoption by these critical guides, if I could have had an opportunity of canvassing their works; but as they have not yet fallen into my hands, you will, good Sirs, excuse me, if I forbear to enter the lists on subjects with which I am little acquainted; for the truth is, the only author smarting under the lash, of whom I can pretend to the slightest knowledge, is my countryman, Mr. Carmichael; and on a comparison of the critique with the work, I have detected so much of mistake, misapprehension, mis-statement, and mis-reasoning, that I have formed a much more favourable opinion than I otherwise wise should of Messrs, Lambe, Arnold, and Reeve, and every other victim of their discipline and abuse.

Had I perused their works, I should probably have selected one of them for my present investigation.--As I have not, I have only to content myself with my countryman's.

Every body knows that Mr. Carmichael has discovered a remedy for cancer, whose efficacy has been evinced by a great variety of instances, in every part of the Empire. The cases published by himself are many in number, and are amply sufficient to satisfy any unprejudiced mind of the powers of the medicine he recommends, so that I can give the Critic but little credit for the affected candour with which he acknowledges, " that Mr. Carmichael's work contains cases of cancer in various parts of the body, cured *apparently!* by the use of iron. These cases are not all very clearly cancerous, but a sufficient number appear to us *incontestibly such*, and in these the *beneficial effects* are *so striking*, as to make us entertain very sanguine hopes that we may be indebted to Mr. C. if not for a specific, at least for the *most powerful remedy* against cancer, hitherto proposed." While, in a note, he attempts to rob him of one half of his merit, because Mr. Justamond, *by chance*, prescribed iron, in a remedy for this disease, along with other drugs, to which he attributed its beneficial effects, without dreaming that he owed its success to the iron; a circumstance with which the candid Critic could not be unacquainted, if it be true, as he insinuates, that he read not only the works of Mr. Justamond, but of Mr. Carmichael, whose unassuming account of the mode of making the discovery, and the progress of his mind in afterwards carrying it on, affords at least, presumptive proofs of his exclusive right to the entire and undivided merit.

But because the evidence of cases arrayed by Mr. C. was irresistable, the gentleman fairly slips them by, and directs his whole artillery against the theory which he finds it easy to represent in a ridiculous light, as indeed it is any theory how well-soever founded.

In my *humble* opinion, (for I am *merely* an Irishman) his attack has ended in utter discomfiture.

Mr. Carmichael does not seem to be " the man of youthful vanity, that fancies himself a genius," according to the brilliant passage already noticed of Mr. Pearson, and quoted by the Reviewer with such fervour of approbation. He appears far from claiming " emancipation from the laws of patient observation and careful induction, which are imposed upon common-sized understandings, nor with a fastidious impetuosity *does he* attempt to rend the veil from nature, by the *mere* powers of his own intellect." He seems to consider, like Mr. Pearson, "that the science of healing, like every other branch of natural knowledge, is not the production of a vigorous imagination, nor a lively invention, but the offspring of long and diligent experience." And he appears to have learned it in the very way prescribed by Mr. Pearson. "In going from his study to the bedside of his patient, and returning from thence to his study again; and if he has penetrated the subtilty of nature, it was by submitting to become her patient and vigilant servant."

Let me however confess, I was at first a little startled at his ideas of "animal fungus," and "independent vitality," but I found the conjecture so strongly supported by a multitude of distinct circumstances, all tending to one point, and corroborating each other, that their connexion was too powerful to be overcome; and in spite of my reluctance, I was convinced of the value of a theory which Mr. Carmichael seems to set very little value upon, satisfied with the more substantial atchievement of establishing the efficacy of his remedy.

Instead of considering each of these insulated facts, and the reasoning founded upon them, our Censor seizes upon one, and that very far from being of the first magnitude, viz. That iron in the blood is, in a certain degree, capable of preventing the production of parasites, and triumphantly asks, "Is it true, that there is in red blood a peculiar salt of iron, which while circulating in the blood, is capable of destroying the life of the simple animals possessing colourless blood? Till this fact is proved, the *whole theory* has not a claim to probability." On looking to the author's disquisition on this subject, I find that he had *previously* given strong facts and reasons for supposing the independent vitality of cancer, as its gelatinous texture resembling zoophytes; its want of connection by blood-vessels with the neighbouring flesh; its insensibility; its appearance in parts endued with little life, or whose organization is injured; its commencement in a point like the incipiency of all animals; its similarity in a variety of respects to hydatids; its increase by roots, and propagation by pea-like substances; and the strange and peculiar pain it occasions. *Afterwards*, when entering on the treatment, he maintains that the remedy itself affords a further presumption of its independent life, and from two simple circumcircumstances, viz. 1st. That oxy-phosphate of iron, exists in the blood, as ascertained by Badia, Menghini, Vauquelin, and Fourcroy; and, 2dly. That oxy-phosphate of iron out of the blood, destroys colourless blooded animals, he infers that there is in red blood, a peculiar salt of iron, capable of destroying the lives of such animals.

Mr. C. having himself ascertained the deleterious effects of iron on worms, &c. by experiments which he calls *coarse*, and which from their nature must have necessarily been so, even in the hands of the most refined and exquisite experimentalist, receives the unqualified approbation of the Critic for dignifying them with that epithet, because in his opinion, as well as Mr. C's, they well deserved it.* But he asks, "Would he not have produced the same effect by corrosive sublimate, tobacco, and various other substances, incapable of curing Cancer?" Certainly he would. And in like manner he could not be ignorant that hemlock is a poison to man, though it will not kill a goat; and that arsenick is destructive both of one and the other; and therefore evinces that there is something in common between men and goats, which hemlock fails to indicate.

"At all events (he proceeds) Mr. C. has proved, that in an undiluted state, a condition in which iron is not found in red blooded animals, that substance is capable of destroying slugs and snails; and having conjectured that cancer is an animal of the nature of earth-worms, &c. he of course infers, that iron will likewise destroy cancer." But this is totally mistaking the drift of the argument, for Mr. C. first proves that iron will destroy worms, &c.; and next, that it has the same effect upon cancer.

* Another cavil on the subject of experiments is, that Mr. C did not ascertain that giving iron internally increased the quantity in the blood; and, above all, did not shew, by chemical analysis, that the blood of cancerous patients contains less iron than that of other persons. No doubt these would be desirable experiments, and since Mr. C. has called the attention of chymists to the subject, we may hope that it will not be long before these facts are ascertained; but they require more time, attention, and precision, than could be well bestowed on them by a person who does not devote his life to chymistry. The Reviewer may suppose that the example of the illustrious Kirwan has made this a fashionable science in the city where he resides; but this is so far from the truth, that it appears from many passages of Mr. C's work, that it is at so low an ebb, that he is indebted to the London chymists for his medicines; and so far from being able to derive any assistance from the few who profess the science in Dublin, that he failed in procuring an account of the analysis of a chalybeate spa, *even* from the Chymical Professor of the Dublin Society.

cancer. Hence he infers that there is something similar in the nature of cancer and worms; an argument of no great strength, but not altogether to be omitted, as it lends some little weight to the conclusion, but totally different however from the deduction that originated only with the Critic.

It is acknowledged, " that Mr. C. has brought in proof of the independent life of cancer, one argument which, if well supported, would indeed have great weight, viz. that the cancerous mass has no connection by communicating vessels with the parts in which it is imbedded. If he is satisfied that the argument is of great weight, I think I shall satisfy him that he has failed in proving that it is not well supported.

Adverting to the dissections by which Mr. C. demonstrates his position, he exclaims, " It is truly wonderful, that in consequence of decomposition, the particles, for example, of a breast or a testicle should so combine as to produce the parasitic animal cancer, and that it should extend in every direction, *sparing only the trunks of arteries*, which were allowed to pass unhurt through the cancerous mass, with the exception of the arterial ramifications blocked up or destroyed. Incredible as this account may seem, such appears the result of Mr. C's dissections. But we strongly suspect that his dissections partook of the coarseness of his other experiments."

The candour that breathes through Mr. C's work, and which seems on one occasion to have touched the Critic very sensibly, is a sufficient assurance that if these were coarse experiments, he would not have concealed the fact; but the Reviewer probably imagines, that our Surgeons of Dublin are dolts and botches, and have neither dexterity, nor information, nor observation, nor intellect; and if such is his belief, he may well imagine the result of Mr. C's experiments incredible, particularly if my critical friend has discovered, as his words seem to indicate, that a breast or a testicle may, by the magic of decomposition, be suddenly transformed into a full-grown cancer, extending in every direction. Such a metamorphose, he might reasonably presume, would *spare* neither trunk nor branch of the blood vessels. But if he cooly considers an incipient cancer, scarcely more than a point, and gradually enlarging in size, he will have no great difficulty to conceive the minute " arterial ramifications blocked up or destroyed" by its progress, while the more powerful " trunks are allowed to pass unhurt through the cancerous mass," progressively growing round them.

He next expresses some doubts, whether the subjects of the foregoing dissections were cancers; but if these doubts are not affected, they will soon be at rest, if he carefully peruses the cases referred to, in which he may satisfy himself (from the history and appearances of the disease before death, and the *carcinomatous substance,* as described by Bailie and Abernethy, found on dissection) that they were genuine cancers. And if he still pretends to doubt, from a dislike to the *scite* of the disease, let him read Mr. C's Disquisition on Lupus and Noli me tangere, at the end of the first series of cases, which he probably passed over with them, in his hurry to grapple with the Author's theory. And if he "will continue to question the truth of the main argument, till it is explained in what manner the independent vitality of Cancer is to be reconciled with the *avowed fact,* that cancer is progagated along the course of the lymphatics," he will perhaps not long continue to question it, if he reads the 4th chapter a little more diligently, in which he will find (p. 300) the *avowed fact* he alludes to, at least greatly shaken if not most satisfactorily disproved and refuted.*

His next objection to Mr. C. arises from his averring, that the breast, uterus, ovaria, and testicles are most subject to cancer at that period of life when their vitality is diminished, and that they are become useless appendages of the system; for though he allows the fact, he somewhat smartly replies, "that the lips, tongue, and stomach are subject to the disease, though they never become useless appendages to the system."

But his short sightedness prevented him from seeing, in *his* very next quotation, a reason why they should also be subject to cancer, viz. "that glandular parts have a tendency to decomposition, as is evinced by their so soon undergoing the putrefactive fermentation after death," a remark, which gives the Critic an opportunity of observing, with great composure, triteness, and gravity, "that the truth is, it is vain to look for analogies between the decomposition of a lifeless mass, and the changes of structure in the parts of a living body. As if no occasion could occur, in which, any elucidation might arise from a comparison of the living body with the dead, or as

* A strong proof of the Reviewer's mistake is, that if an animal receives a bruise before it is killed, the part affected is the first to run into the putrefactive fermentation afterwards. We have no instance of cancer observed in cattle, but in human beings a bruise is the most common cause of the disease.

as if the different degrees of vitality were to be allowed no zero in the standard that measures them.

But garbled and divellicated from their meaning and mutual support, as Mr. Carmichael's arguments appear in the critique, the objections brought against them make but little impression on a person, who carries in his mind the connected series in which he has unfolded them. I cannot well quote one observation, without others relating to the subject in question, and shall barely refer the reader to the passages contained between pages 262 and 273; and from the information I have there obtained, shall proceed, without any quotation, to disentangle the skein of difficulties by which a very plain story may be rendered obscure.

The Reviewer asks, if the rapidity of decomposition be the cause why cancer does not take place when mortification occurs, how are we to account for the absence of cancer in that *slow* dissolution or gangrene of the extremities occasioned by the ossification of the arteries? I answer, such a gangrene is a real mortification; and the dissolution is not slow, but quick. It may be slow, with respect to the entire limb, but it is instantaneous with regard to that portion which the blood ceases to visit.

And again, "If a diminution of life be the cause of that tendency to dissolution and actual decomposition, which gives rise to cancer in the breasts of old maids, and in the testicles of old men, is there not still less activity and life in paralytic limbs, and therefore a still more powerful cause of cancer?" I reply, there is less *activity* in such limbs from the affection of the nerves, but not less *life*; because the circulation, though more languid than in health, is more powerful than in the testes or mamma alluded to, and therefore paralytic limbs are less liable to cancer.

"If it be true, that the cause of cancer is a diminution of life connected with a languid circulation, the indication of cure should be to excite the drooping powers of life by stimuli, so as to accelerate the arterial action and prevent decomposition; a practice contradicted by the most eminent writers and by Mr. C. himself." This query strongly corroborates Mr. C's theory, for if the natural indication of cure is more injurious than serviceable, it follows, that the disease is radically and essentially different from every other—and why does not accelerating the arterial action cure the disease? Because it is an animal fungus unconnected with the arterial system, and though though it might, perhaps, have been *prevented* by such acceleration, it can never, by that means, be cured.

The same fact accounts for the beneficial effects of lessening the force of the circulation after the disease is formed, for " heat and moisture are produced by the increased force of the blood and action of the vessels, than which nothing can more conduce to the growth of parasites. Pain and irritation must at all events be the consequence around the extraneous body, whether living or dead ; and it is natural for the patient to think, that whatever produces pain must be injurious. Yet, even this admits of a doubt, as the very irritation he complains of, may be the fore-runner of ulceration, an event that checks the progress of the disease, and is an effort of Nature to throw it off."—p. 392 of Essay.

But *before* the formation of the disease, the lowering system would evidently be no trivial auxiliary to its production ; and it is a well known fact, that women who suffer most from menorrhagia, are most subject to carcinoma.

The Reviewer objects against the observation, that suppuration only takes place when a portion of the cancer loses its vitality, as in the Guinea-worm ; that it might as well be said, that the suppuration of a scrophulous gland depends upon the same cause. This I acknowledge, if the position stood *singly* ; but if it has been previously proved, that cancer is an animal fungus, this argument corroborates the opinion, though it could not have verified it.

With respect to the fact, that cancer sometimes assumes the hydatid structure, the Reviewer makes some merry observations, and entertains an ardent, and indeed a very witty hope, that it may continue to improve, till it exerts its locomotive powers in walking out of its human habitation. But the world is pestered enough already with walking *parasites* ; and I am sure, Messrs. Davy and Pearson will join me in the wish, that there was rather a truce to the compliments of those already *in esse*, than that there should be an increase to the brood.

The passage contains no objection to be answered; and I have only to remark, that he is under a mistake with respect to Dr. Adams's opinion ; for that Gentleman does not suppose the cavities usual in cancer to be hydatids, but the cells containing fat, which are formed by the roots of the cancerous mass.

From this part of his critique, as well as from the *in-*

*comprehensible*

*comprehensible* train of fancied reasoning with which it begins, the writer appears hostile to every attempt, tracing any laws concerning the production and powers of those beings which form the lowest links of life, and almost to insinuate, that it would be sinful to advance a conjecture as to the means by which they entered into existence; as if the equivocal birth of a parasitic mushroom or intestinal maggot, could affect our long established opinions of the creation of man and other animals, whose mode of generation is obvious. But with respect to his sneer at the Reviewers of Darwin and Hunter, let me ask him, if a new-laid egg does not possess the living principle?—if not, why should it bring forth a chick under the warmth of a hen, when a stale one, in which that principle is incontestibly extinct, will never be hatched? And if he acknowledges the fact, is not a fresh egg an instance "that life may exist without matter being in a state of action, and only evinced by the property of self-preservation preventing decomposition." Let him next account for the production of the green vegetable matter noticed by Priestley; for that of hydatids in the livers and brains of sheep*; of worms, whose genera no where exist but in the intestines; and particularly, let him explain how a worm could have found a way into the intestines of an infant in the womb. If he can do all this, without departing from his enmity to spontaneous and equivocal generation, I shall willingly admit my own igorance, and that of Messrs. Hunter and Darwin, and my friend Carmichael.

In the only observation remaining to be considered, our Critic, arguing on the benefit arising from the internal use of iron, exclaims, "We cannot *see* how, according to the theory, an increased quantity of iron in the blood can have any effect in destroying cancer, since a vascular connexion between the diseased mass and the parts around, is denied by the author." But if carcinoma, in drawing its nourishment, by its own intrinsic powers, from the surrounding parts, and with it imbibes a quantity of the iron, may it not thus be destroyed, although there should be "no vascular connexion between the diseased mass and the parts around?"

The

* The latter fact may evince the tendency of the living brain to run into decomposition, and *accords* with the Reviewers remark, that after death it undergoes the most rapid putrefaction.

The Critic must have had some ingenuity to pass over his author's remarks on the office which iron performs in the circulation. He exhibits no less contrivance in shutting his eyes to the more useful observations of Mr. Carmichael, in which he discriminates the two species of consumption, which require such opposite modes of treatment, that what may cure in one will kill in the other. This momentous discrimination is not even hinted at, except in an observation on Mr. C's description of tubercles, as bearing "a resemblance to cancer of the face, in their size, appearance, and structure, and the mode in which they produce ulceration and suppuration of the surrounding parts,"* *which does appear* to the Critic "so preposterous, and so far from the truth, that he must deny the allegations altogether."

After this acute reasoning, and perspicuous criticism, follows the sentence from which alone we can learn any thing of Mr. C's discrimination of two species of consumption, "The plan of treatment grounded on such a theory, is obviously to be the opposite of that recommended in the florid consumption."

Other diseases demonstrated by the author to arise from too much, or too little, iron in the system, were also unworthy of a remark. But why should he bestow a thought upon trifles when to have brought to light an alleviation, and, in some cases, a remedy, for the most gloomy and stubborn of all diseases. When, to have promulgated it to the world the instant its value was ascertained. When, to have detailed its virtues and defects, and the various modes of promoting the one, and guarding against the other, with a minuteness and precision that puts every *willing* practitioner on a level with the inventor, in prescribing and administering every preparation he employs. When, to have done all this, of which not a particle is denied, obtained no better treatment than that which I have just investigated. But when we compare it with the praises lavished on a chapter, however valuable, on the same subject, containing only an imperfect attempt at the discrimination of diseases, and an honest lamentation that the author can afford them no cure or palliation, we shall leave our readers to make the inference.

Few men who consider the matter in the light I have done, will not be disgusted with the strong features of grimace

* Perhaps Mr. C. alludes to the description of cancer of the face, p. 103.

grimace displayed on all occasions by this Reviewer. If he has any regard to the improvement of a science, the most important in this life, let me seriously recommend him to peruse the subjects of his criticism a little more at large, and with a little more industry, if he wishes to comprehend them; and a little more candour and fair dealing, if he expects any deference to his decisions. He will find in the end, if he has not already found, that it is not possible to write down a work of merit, or impede the progress of improvements founded in reason. T. R.

---

The perusal of the above will suggest to our Readers, the illiberality of attempting to crush, as it were, every improvement in the bud. Who will venture to offer a remedy, however valuable, to the public, if the consequence is to be a fastidious and unfeeling attack on every vulnerable part of his publication? And what so easy as to make a thing ridiculous? The great object of good breeding and a liberal education is to correct that propensity to satire, always met with in vulgar minds. This is so well known that men are ashamed to commit their names to such publications. But does not this argue something worse than we have expressed?

It is true, that works of diligence, accuracy, and information have found their way, in spite of the most artful attempts to arrest them. But these are not the only considerations; when the work of a physician, who, the Reviewers *are informed* is a young man of much industry and *some* talent, is thus exhibited to a neighbourhood, in which he expects his future advancement, it is impossible to say what advantage may be made of it by an unprincipled opposition. The critique is exposed to the view of those who cannot understand the subject, and the force of ridicule is often irresistible.

These suggestions have been strengthened in our minds by a correspondence which the recommendation of cob-web has produced. Can we expect to receive useful practical information, if we expose those who favour us with it to all the severity of ridicule? We have unquestionable authority, that an ague in Walcheren, which resisted bark and arsenick, was cured by cob-web, even in the pestilential air from whence it arose. Can we then admit the wit, however classical or refined, if directed against those who would direct our attention to such a remedy?

